# Perspectives of healthcare providers on osteoporosis, falls and fracture risk: a systematic review and thematic synthesis of qualitative studies

**DOI:** 10.1007/s11657-024-01446-8

**Published:** 2024-09-24

**Authors:** Catherine Cho, Grace Bak, Daniel Sumpton, Bethan Richards, Catherine Sherrington

**Affiliations:** 1 Faculty of Health and Medicine, School of Public Health, The University of Sydney, Sydney, Australia; 2 The Institute of Musculoskeletal Health, Sydney, Australia; 3 Concord Repatriation General Hospital, Sydney, Australia; 4 Royal Prince Alfred Hospital, Sydney, Australia

**Keywords:** Healthcare, Osteoporosis, Fracture, Falls

## Abstract

**Objective:**

Osteoporosis and falls are major risk factors for osteoporotic fractures, with significant detriment to patients’ quality of life. We aimed to describe healthcare provider (HCP) perspectives and experiences in the diagnosis, management and prevention of osteoporosis, falls and fractures obtained through primary qualitative research.

**Methods:**

Thematic synthesis was performed on articles identified through a search of electronic databases (MEDLINE, Embase, PsychINFO and CINAHL), which were searched from inception to May 2023.

**Results:**

Twenty-seven studies including 1662 HCPs, including general practitioners (GPs), physicians, surgeons, physiotherapists (PTs), occupational therapists (OTs), pharmacists and nurses, were included, with identification of six themes: overshadowed as a disease entity, uncertainty in decision making, frustration with interdisciplinary and systemic tension, avoiding medical paternalism, desire for improved care and embracing the responsibility.

**Conclusion:**

Osteoporotic fracture and fall prevention in routine clinical care is hampered by inadequate priority and lack of perceived connection with morbidity and mortality, deficits in interdisciplinary collaboration, lack of clinical confidence and health resourcing. However, HCPs acknowledge their role in promoting healthy ageing, thus providing support through appropriate continuing education, resourcing and public health campaigns that are significant future directions, which may improve osteoporotic fracture prevention.

**Supplementary Information:**

The online version contains supplementary material available at 10.1007/s11657-024-01446-8.

## Background

Osteoporosis and falls are independent major risk factors for fractures. The lifetime risk of osteoporotic fractures for women and men ≥ 60 years is approximately 44% and 25% respectively. There is recognition of increased mortality with all fracture types, up to 20% at 12 months for hip fractures, with fractures imposing significant physical and psychological burdens on individuals and broader societal financial implications [1–3]. The global burden of low bone density is increasing, with an attributable rise in global deaths by 111.16% to 437,884 and disability-adjusted life years (DALYs) by 93.82% to 16,647,466 between 1990 and 2019 [4].

Despite the availability of effective osteoporosis pharmacotherapy and evidence-based fall prevention programs, there remains a significant gap between best practice recommendations and real-world management [5–10]. Qualitative research is a form of ‘social inquiry’, which aims to describe the ‘how’ and ‘why’ of human behaviour, which is difficult to quantify through quantitative methods. Qualitative research methods have been used to describe the attitudes, perceived barriers and knowledge deficits that may help explain the disparity between guidelines and real-world clinical practice. A systematic review and qualitative synthesis can summarise and extend qualitative research to create new themes and understanding of barriers that may help to inform better healthcare delivery. A systematic review of the implementation of fall prevention programs found success of interventions required modification of established practices, behaviours and thoughts [11]. Thus, the aim of this study was to describe healthcare provider (HCP) perspectives and experiences in the diagnosis, management and prevention of osteoporosis, falls and fractures obtained through primary qualitative research. This would provide in-depth insight into clinical decision making which may inform the design of future interventions to reduce the burden of osteoporotic fractures.

## Methods

The Enhancing Transparency of Reporting the Synthesis of Qualitative Research (ENTREQ) framework was utilised to design the study protocol [12]. The study protocol was registered on PROSPERO (CRD42022331962).

### Selection criteria

Primary qualitative studies on the experiences, perspectives and attitudes of HCPs, including medical practitioners (general practitioners, physicians, surgeons), allied health (physiotherapists, occupational therapists, podiatrists), pharmacists and nurses, on osteoporosis, falls and/or fracture management and prevention were eligible for inclusion. Quantitative studies, abstracts, reviews, commentaries and letters were excluded. Mixed-method studies reporting quantitative and qualitative data were only eligible if qualitative data could be extracted separately. Non-primary qualitative studies such as reviews, clinical guidelines, quantitative surveys, studies on the acute management of osteoporotic fractures and non-English articles were excluded.

### Data sources and searches

Electronic databases MEDLINE, Embase, PsychINFO and CINAHL from the date of inception to 24 May 2023 were searched (Online Resource 1). Two authors (CC and GB), reviewed all references, with duplicates and studies not meeting inclusion criteria excluded. Reference lists of relevant studies were hand-searched for additional primary studies for inclusion.

### Comprehensiveness of reporting

The comprehensiveness of reporting was assessed using the Consolidated Criteria for Reporting Qualitative Health Research (COREQ) framework [13]. The framework includes reporting items related to the study methods, research team, data collection, analysis and reporting. Two authors (CC, GB) independently assessed studies using the COREQ framework. A third reviewer (DS) adjudicated any disagreements.

### Synthesis

Data was analysed using inductive thematic analysis, using a qualitative description approach [14, 15]. Articles were analysed line-by-line following extraction of quotations, results, discussion and conclusion into Nvivo Ver 14. Authors CC and GB independently read each article line-by-line and coded text into inductively derived concepts. Concepts were grouped into themes and subthemes, which were discussed with the research team and then refined in an iterative process to ensure researcher triangulation. Conceptual links were identified to develop a thematic schema.

## Results

### Literature search

**Fig. 1:**
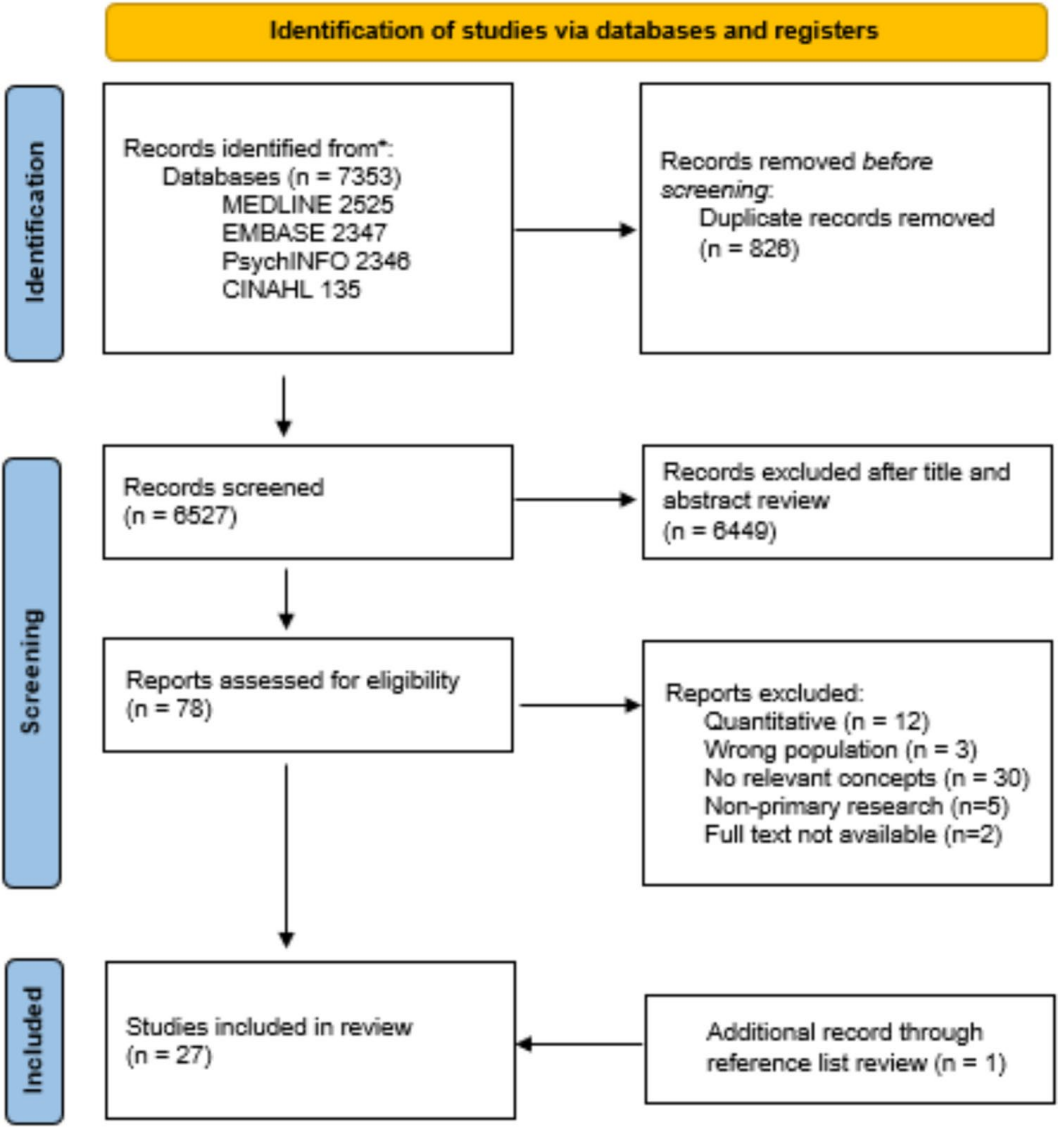
Database search

The database search yielded 7351 studies, with 26 studies meeting the inclusion criteria. One additional study was identified from hand-searching reference lists (Fig. 1). A total of 27 studies were included, with a total of 1662 participants. Study characteristics are outlined in Table 1. This number included two mixed-methods studies which included questionnaires with open-ended questions. There were 521 participants from studies utilising semi-structured interviews and focus groups. HCPs included physicians (primary care, endocrinologists, rheumatologists, geriatricians, gynaecologists, oncologists), general practitioners (GPs), orthopaedic surgeons, allied health professionals (physiotherapists, occupational therapists, podiatrists), pharmacists and nurses. Full characteristics of studies are presented in Online Resource 2.

**Table 1:** Study characteristics

Year of publication	Number (%)
Region	
Australia	6 (22.2)
UK	4 (14.8)
North America	10 (37.0)
Europe	6 (22.2)
Other	1 (3.8)
Sample size	
1–20	17 (63.0)
21–40	5 (18.5)
41–60	3 (11.1)
> 60	2 (7.4)
Topic	
Osteoporosis	17 (63.0)
Falls	7 (26.0)
Fractures	3 (11.1)
Method of data collection	
Interview	17*
Focus groups	12
Other	3
Healthcare provider	
Doctor	1489 (90.0)**
GP/PCP	1293
Other	196***
Nurse	82 (4.9)
Allied health	38 (2.3)
PT	22
OT	16
Pharmacist	20 (1.2)
Other, not specified	33

*GP* general practitioner, *PCP* primary care physician, *PT* physiotherapist, *OT* occupational Therapist

*Four papers utilised focus groups and interviews

**A total of 1054 from [16] (survey) and 87 from [17] (survey with open-ended questions)

***Primary care, endocrinologists, rheumatologists, geriatricians, gynaecologists, oncologists

### Comprehensiveness of reporting in included studies

A modified Consolidated Criteria for Reporting Qualitative Health Research framework (COREQ) was used to assess the reporting of interview and focus group studies (Online Resources 3 and 4). Two studies utilised mixed methods, with open-ended questions in surveys used; thus, COREQ was unable to be applied. The comprehensiveness of studies was variable (range 24.1 to 75.9% of COREQ criteria met). Illustrative participant quotations were reported in 25 (92.6%) of the studies. Nine (33.3%) of studies documented data saturation. Researcher triangulation was described in 17 (63.0%) of the studies.

### Synthesis

**Fig. 2:**
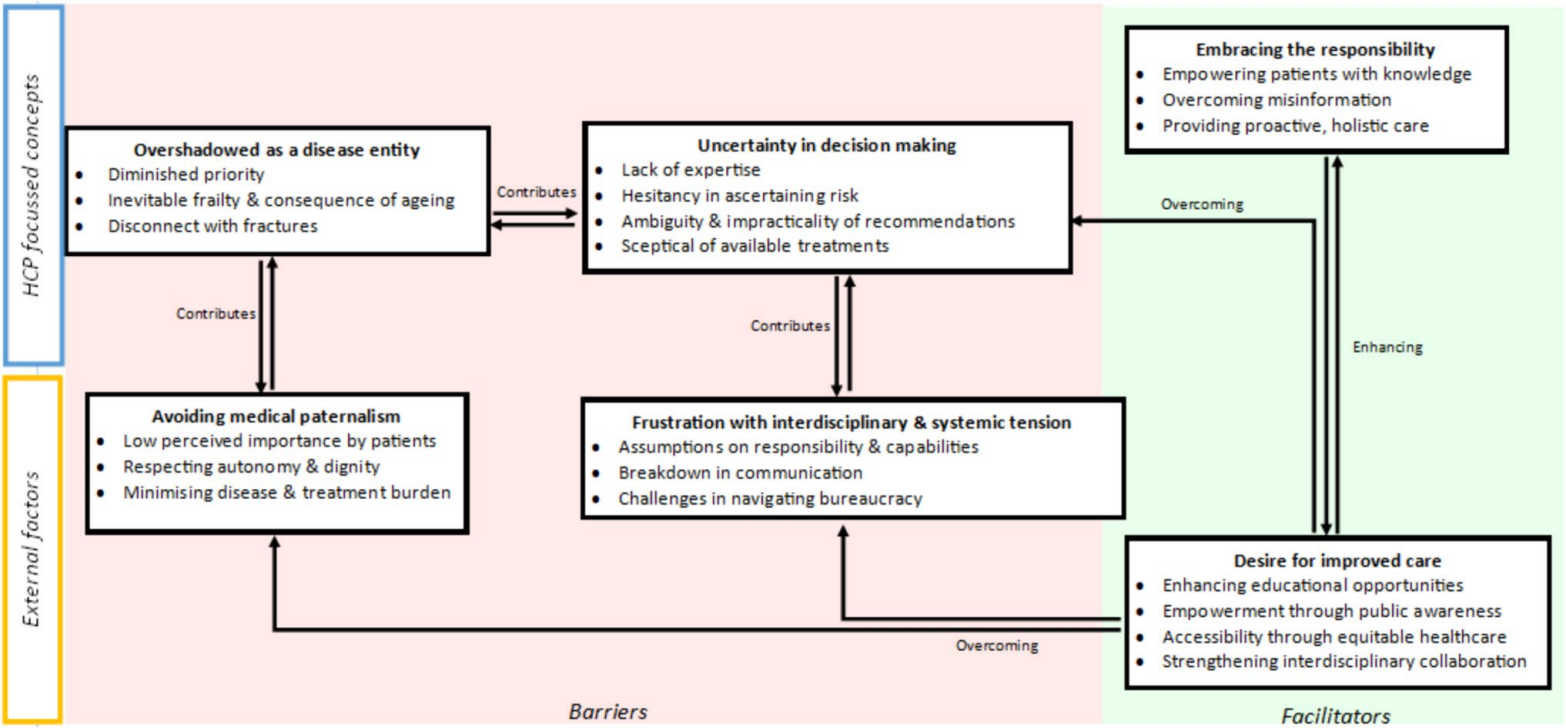
Thematic schema. HCPs faced challenges prioritising osteoporosis, falls and fracture prevention and treatment amongst the increasingly complex landscape of multi-morbidity within constrained healthcare systems. This was compounded by varying levels of clinical confidence within this field and a desire to respect patient decisions to promote therapeutic relationships. There were increasing levels of recognition of its relevance, particularly with the ageing population, with HCPs longing for trusting relationships with other providers to provide holistic care, supported by educational resources and increasing public awareness

We identified six themes: overshadowed as a disease entity, uncertainty in decision making, frustration with interdisciplinary and systemic tension, avoiding medical paternalism, desire for improved care and embracing the responsibility. Illustrative quotations are provided in Table 2, with the relationships mapped in a thematic schema (Fig. 2). Most HCPs were GP/PCPs; however, similar perspectives and experiences were shared between different HCPs. If differing perspectives were present between different types of HCPs, these have been delineated in the synthesis.

**Table 2:** Illustrative quotations from primary studies

Theme	Subtheme	Illustrative quotations	Contributing studies
Overshadowed as a disease entity	Diminished priority	GP: ‘There is something insidious about it as it slowly worsens. It’s not a cancer that can kill you in 6 months and metastasize.’ [19] GP: ‘A lot of my patients have COPD and they are coming in with an exacerbation... or they are coming in with their blood pressure poorly controlled.’ [32] GP: ‘I will not speak of OP systematically, far from it! because I already have so many other things to talk about, diabetes, and smoking, and alcohol, and...’ [20] GP: ‘Well, none of these preventative things – they don’t help you, they only really help you if you want to take it for years. There’s absolutely no rush whatsoever in convincing anything like this. Like blood pressure medication, nothing is going to happen next week if you don’ttake it.’ [38] GP: ‘I think that patients with osteoporosis that often becomes manifestin old age…those patients often have significant comorbidity that contributes to making the problem [osteoporosis] invisible.’ [18]	[18–21, 23, 25–27, 29–38, 70–72]
	Inevitable frailty and consequence of ageing	GP: ‘In my view, it’s not a disease, in that it’s the aging process, and we will all be osteoporotic.’ [19] Nurse: ‘As far as the boat having sailed when you see an elderly female patient..’ [29] GP: ‘first I have to accept, and I haven’t done it yet, that OP is something other than normal progression.’ [20] GP: ‘they are afraid of aging... one falls, fractures, then we are in bedridden, then the hospital, and then the person dies! It’s aging... the disease itself, called osteoporosis or natural aging, I do not think they distinguish!’ [20]	[17–21, 29, 72]
	Disconnect with fractures	GP: ‘You look at cases of vertebral compression, but there are so many vertebral diseases now that I’m not sure you can really say that there’s a link.’ [19]	[19, 20, 37]
Uncertainty in decision making	Lack of expertise	GP: ‘To know with which one to start, when to switch, because we will have a treatment for 20 years, it is not easy… we did not really have a lot of information on it, and it’s not so easy to prescribe.’ [20] Pharmacist: ‘I’m not that confident because I do not have a big picture of what is the treatment, what is the medicine.. so the management part, I am not that familiar.’ [37] GP: ‘Unbelievably confusing about who to treat with what and for how long!’ [16] GP: ‘Duration of bisphosphonate therapy appears to be the Wild, Wild West of osteoporosis treatment… no one seems to know the optimal duration of bisphosphonate therapy.’ [16]	[16, 18–20, 23, 27, 29, 36–38, 72, 73]
	Hesitancy in ascertaining risk	GP: ‘… we do not necessarily have a correlation between fracture frequency and BMD results, therefore what is right? Do we have the right marker?’ [20] GP: ‘…difficulties in interpreting the figures, T score, Z score… and FRAX: I calculate it on the internet, I have a percentage, but I do not know what to do? It is said ‘it’s great you have a percentage risk of, yes but so what?’ [20] GP: ‘I’m not sure what business we have of prognosticating 10 year risks on people over the age of 80. 10 year risks are ridiculous. Nobody knows what’s going to happen 10 years down the line.’ [25] GP: ‘The high-risk and low-risk patients are easy.. it’s those darn moderate risk patients and trying to determine who require(s) treatment.’ [16]	[16, 17, 20, 24, 25, 27, 29, 38, 72, 73]
	Ambiguity and impracticality of recommendations	GP: ‘in reality, osteoporosis is a complex issue.’ [27] GP: ‘There’s just so much noise in the system you now, and you’ve got NICE* saying one thing and then you’ve got NOGG* coming along and saying something which seems, eminently actually to me more sensible and more straightforward, maybe not as rigorous.. so I think if you could get one message, because when there’s one message, it’s simple and it’s clear.’ [26] GP: ‘I felt it the study came out with some clearer guidelines for us then it would be helpful for everybody.’ [26] GP: ‘I think that there are sort of conflicts in having guidelines that are actually useful in making an individual treatment decision whilst being sufficiently simplistic enough for someone to be able to commit to memory and remember.’ [28]	[17, 20, 25–29, 33]
	Sceptical of available treatments	GP: ‘Some patients won’t tolerate the alendronate** very well. I’ve had a bunch that just can’t take it.’ [29] GP: ‘I have some major philosophical problems with both the diagnosis and treatment of osteoporosis, where do you draw the line of risk and benefit? The majority of people on therapists… will not do them good.’ [29] GP: ‘I sometimes wonder about the cost benefit. You know, in terms of how many fractures are we really saving? How many people do we have to treat to save, you know, a fracture? You know, if we were preventing the fractures in the fifty to sixty year old, I’d say that’s a bloody good thing but most of these fractures occur in people who are at the end of their lives and the sort of fractures that are more common, like the vertebral fractures, well, people don’t die from that.’ [72] GP: ‘… side effects! For a benefit that has not really been proven! I think there’s more osteonecrosis of the jaw with long lasting treatment.’ [20] GP: ‘I’m a bit reluctant to start medication unless it’s really indicated because of the potential side effects. Mostly the rare bone fractures and other indigestion type sort of problems.’ [38]	[17–20, 27, 29, 30, 35, 36, 38, 72]
Frustration with interdisciplinary and systemic tension	Assumptions on responsibility and roles	Doctor: ‘I’d rather have them back and do it myself..’ [22] GP: ‘Who’s managing this? Do you want me to manage it or not?… I think that’s part of the issue.’ [22] FLS Doctor: ‘We took it out of the hands of GPs because it was being done so poorly…’ [22] FLS Doctor: ‘It’s almost like [GPs] need a package of almost instructions with a tick box… so that yeh it’s straight forward for them.’ [28] FLS Doctor: ‘This has to be owned by Primary Care… GPs are responsible for managing long term conditions.’ [28] Nurse: ‘I think we all know it [falls education], and we just leave it to the physios to do.’ [31] OT: ‘I think that probably they don’t realise the scope of what we do half the time – I think there’s been a history of the equipment providers and that’s all we are.’ [30] GP: ‘if there’s a manifest fracture, then I think that you can also require the hospital to help us.’ [18]	[18, 20–23, 28–31, 33, 36]
	Breakdown in communication	GP: ‘It takes a while, there is delay, it usually takes a month.’ [22] FLS Doctor: ‘I spend so much time generating letters and I sometimes wonder at the wasted time. I’d really like some metrics on… effective communication..’ [22] FLS Doctor: ‘GPs get probably 400 or 500 letters a day, do they read everything? Hopefully they do.’ [28] GP: ‘Some people have had one and they don’t know what the result is.. then you might have to do a little searching.’ [29] GP ‘… maybe 5 or 6 months, or maybe never, they come back to see us. By that time, [the fracture] has sort of faded into the background…’ [29] GP: ‘I wouldn’t know about the results unless I, for whatever reason, actively set out their results.’ [29] OT: ‘Physiotherapists have gone off on their own tangent and not come back and had mutual discussions..’ [30]	[18, 21–23, 28–31, 71]
	Navigating care with insufficient resources	GP: ‘Fall prevention might take a long time and you don’t get reimbursed for any of the fall prevention counselling you do.’ [32] GP: ‘Our biggest barrier is obviously the fact we have to drag our patients from another town down to the city’ [28] GP: ‘Sometimes you have to be a bit liberal on your ticking…’ [26] GP: ‘The other thing patients definitely do for cost issues, is split the doses.’ [71] Oncologist: ‘I don’t know where I would refer the patient if they were having some frailty falls.’ [34]	[17, 18, 21, 22, 24, 26, 28–30, 32–34, 36, 71, 72]
Avoiding medical paternalism	Low perceived importance by patients	GP: ‘They are not going to report, oh well I fell. Our patient population does not see it as a big thing.’ [32] GP: ‘More pressing issues – no time for disease prevention.’ [27] Pharmacist: ‘So far, we don’t really have any customer that comes to the pharmacy and say, I want to prevent osteoporosis.’ [37] GP: ‘Patients seldom come and visit us to discuss osteoporosis. And then it happens easily that you don’t think of it either.’ [18]	[18, 20, 27, 29–34, 37, 38, 71, 72]
	Respecting patient autonomy and dignity	GP: ‘These are not patients who are going to admit that they are losing mobility, because they think the next step is a nursing home.’ [32] PT: ‘They’re not wanting to give up their independence.’ [31] OT: ‘And they don’t want to use those because they think they’re not old enough for aids and those types of things.’ [31] Oncologist: ‘Most of them are dismissive of it… they’re embarrassed, or the word about being frail and not being able to function independently… especially when their caregivers in front of them.’ [34]	[20, 21, 23, 29, 31, 32, 34, 38, 70]
	Minimising disease and treatment burden	GP: ‘I am sure there will be some people who were going along feeling perfectly healthy as with anything like that and then they suddenly find they’ve got a label and it’s a bit worrying.’ [26] GP: ‘The people interested in osteoporosis are the group of women who are between 55 and 65 years of age, they are very interested I health… telling them, ‘Oh, well, we don’t really check this – it’s too early to do it,’ just doesn’t wash very well with that group.’ [29] Physician: ‘It is nothing to worry about. Most of those women will never experience a fracture. I think that most of them would live a happier life without knowing that their T score is lower than 2.5.’ [35]	[26, 27, 29, 32, 35]
	Negotiating patient acceptance	GP: ‘You often establish relationships and people can become more trusting and they can tell you if they’re taking their medications or not.’ [28] PT: ‘I think you know when patients aren’t doing exactly what you want them to but.. it’s really, um letting them..’ [23] PT: ‘The campaign’s very much like a very broad brush that’s used… the next campaign it shouldn’t take that approach, it should be based on what’s needed for that particular patient.’ [31]	[18–24, 28, 29, 31, 33, 35, 38, 70, 72]
Desire for improved care	Enhancing educational opportunities	Nurse: ‘Yes, we want to know. New things and new research come along all the time.’ [36] Nurse: ‘I would definitely go on a course like that.’ [36] GP: ‘I need to increase my own knowledge.. how many fractures can you prevent? How effective are those drugs and what kind of side effects do they have? What is the cost benefit for patients?’ [18] GP: You know, some ofthe things that might help as well is to have a significant education outreach and to have information that can be passed to the patient—that might be a benefit [29] Doctor: Some time in my third year of residency we got a crash course in geriatrics. And the thing that really stuck with me was if they break their hip they have a huge mortality [32]	[18, 20, 25, 27, 29, 32, 33, 36]
	Empowerment through public awareness	Nurse: ‘… and everyone really, media as well – should know about such as widespread disease. It’s so important to get the word out.’ [36] Pharmacist: ‘If there is a public campaign, and then concurrent together (with) pharmacies, then we will be able to get the numbers that we hope for..’ [37]	[18, 20, 22, 36, 37]
	Accessibility through equitable healthcare	GP: Our biggest barrier is obviously the fact that we have to drag our patients from [another town] down to [the city]. [28] PT: Our main barriers would be geographic – it is a dispersed population on the west coast of Ireland and the road network is not good and there are a lot of older people living in isolated farmhouses or poor housing conditions [30] PCP: We try to get them into whatever program is either closer to where they live or within the scope of their dial-a-ride... So it is not that we are not referring, but they do not have the means of getting there and getting back [32] GP: If you’re working in a practice where your minimum appointment is six minutes, which is generally terrible medicine, which is what you see in some of the clinics in the area [22]	[22, 26, 30, 32]
	Strengthening interdisciplinary collaboration	Physician: ‘It’s just about encouraging people to know that they all have a role, we all have a responsibility to deliver quality care and all of us are important in making that so. You know each of you can’t do it without the other and it’s actually about ownership and responsibility.’ [28] Surgeon: ‘If we really have our best interests in mind for our patients in reducing hip fractures, then we are going to have to figure out a way to collaborate with primary care.’ [29] GP: ‘My dream would be to have a couple of hours a week set aside for team consultations.. and we could identify where there were gaps and overlaps. Everyone could pool their ideas about what would be best.’ [21] GP: My main role would be to detect, identify those patients and if there are obvious medical issues that I can help them with to improve their general mobility and so on. But beyond that, identify specific people to help patients in their physical environment [21] GP: I had an extremely poor understanding of what an occupational therapist did until I started working with an occupational therapist...like their cognitive assessments and things that I’d never have thought of [30]	[18, 21, 22, 28–31, 36]
Embracing the responsibility	Empowering patients with knowledge	PT: ‘More of an interactive coaching interview, you know how you were saying that people come up with the ideas themselves they’re more likely to implement them or be responsive to them..’ [31] GP: ‘It is our role to explain.. if they (patients) do not really understand what OP is, I think that they will not accept treatment.’ [20] GP: ‘I think if they can understand what they’re trying to prevent and how it can impact on their lifestyle, the loss of independence is a major motivator to keep the treatment up.’ [38]	[18, 20, 22, 24, 27, 29, 31, 33, 35, 38]
	Overcoming misinformation	GP: ‘We (the GPs) have knowledge about the conditions and we can spot which problem the patient has. Sometimes, I tell them that it is nothing to worry about and then they trust me and stop being worried. That is my role as a GP. I have seen many patients and thus have a huge medical knowledge about diseases, because of my 20 years of experience. The patients do not have this knowledge, and they cannot achieve that just by spending some time on the Internet.’ [35] GP: ‘They are getting all this information from the media… You shouldn’t be taking this… Everything that comes out of the news people listen to and then call their doctor and say, ‘should I be taking this?’ And [they] start doubting whether they should be on it.’ [71]	[20, 29, 35, 71]
	Providing proactive and holistic care	Nurse: ‘The age-75 discussion affords great opportunities. Then you really go through everything. What they eat and drink and how much or how little they exercise and everything.’ [36] GP: ‘To actually have a fracture is quite an impact on someone’s morbidity and indeed mortality isn’t it, you know lots of little old ladies... perhaps [do] not survive having a fractured hip actually so to try and pick them up say five years earlier and to try and reduce the amount of bone density loss is certainly going to be a good thing in my view.’ [26] GP: ‘We should consider OP, because there is an aging population and we have to prevent degradation of health in the very elderly.’ [20]	[20–23, 25, 26, 31, 36, 72, 73]

*GP* general practitioner, *PT* physiotherapist, *OT* occupational therapist, *FLS* Fracture Liaison Service

*NICE (National Institute for Health and Care Excellence), *NOGG* (National Osteoporosis Guidelines Group)

**Changed to generic name from originally quoted trade name

## Overshadowed as a disease entity

### Diminished priority

The ‘invisible’ nature of osteoporosis and the lack of perceived association with morbidity and mortality led HCPs to overlook osteoporosis and falls in routine clinical practice [18]. Compared to other co-morbidities, such as ‘diabetes, smoking and alcohol’, osteoporosis and falls were perceived as ‘not what’s going to kill my patient [19, 20]’. HCPs were thus more likely to prioritise secondary over primary prevention. Whilst some HCPs recognised the importance of primary preventative care to reduce osteoporotic fracture risk, they felt overburdened due to time constraints in the context of other priorities.

### Inevitable frailty and consequence of ageing

Moreso than physicians, GPs, gynaecologists and surgeons described osteoporosis and falls as ‘the ageing process’ and ‘frailness’, with falls and fractures as ‘inevitable’, rather than a disease or process amenable to prevention [19, 21]. The asymptomatic nature of osteoporosis reinforced this belief. This sentiment extended to treatment decisions. GPs had a pervasive, pessimistic attitude towards both osteoporosis pharmacotherapy and fall prevention programs, with some considering the elderly as ‘too old’, or having a ‘short-life expectancy’, to warrant management [17, 18].

### Disconnect with fractures

Several HCPs disassociated fractures with osteoporosis and falls, a perception particularly prevalent amongst orthopaedic surgeons. Whilst most eventually realised the relationship, it became apparent only after a fracture had occurred. HCPs additionally felt patients conceptualised falls and fractures as a transient consequence of an accident or risky behaviour, rather than reflecting underlying pathology. This directly contributed to perceived disinterest in medication initiation from patients, medication non-adherence and resistance to fall interventions, both adding to the challenge of delivering care.

### Uncertainty in decision making

#### Lack of expertise

Some HCPs, particularly GPs, felt osteoporosis pharmacotherapy was ‘not so easy to prescribe’ and felt unsure about the length of treatment duration and monitoring, causing hesitancy in prescribing [20]. Providers lacked confidence prescribing in the co-morbid elderly patient, regarding duration and choice of therapy and managing patients who fractured on anti-resorptives [20]. Specialist physicians, including rheumatologists, were more comfortable with pharmacotherapy [19]. Some physicians working in Fracture Liaison Services (FLS) voiced doubt in GPs’ knowledge of osteoporosis treatment [22]. Limited knowledge extended to the provision of fall prevention information, particularly amongst doctors compared to nurses and allied health practitioners [23]. HCPs felt these deficits in knowledge made it difficult to provide adequate patient education and initiate appropriate referrals. GPs, nurses and pharmacists all criticised the low coverage and lack of specificity of osteoporosis management during training and absence of suitable educational material reflecting evolving literature.

#### Hesitancy in ascertaining risk

Although GPs were confident in identifying high-risk patients, they found certain patient populations challenging, including men and pre-menopausal women. They had dilemmas in identifying ‘who to throw into the system at the onset’, with DEXA and pharmacotherapy, due to concern for overdiagnoses and treatment [24]. There was also distrust and doubt of the clinical significance of risk assessment tools and DEXA scans, due to variability in reporting and inconsistency in inclusion of risk factors. The 10-year risk prediction from assessment tools was considered ‘ridiculous’, particularly in the elderly in which prognosis was perceived as limited [25].

#### Ambiguity and impracticality of recommendations

Confusion with differing recommendations between multiple guidelines was shared amongst HCPs, particularly GPs. They felt guidelines were not applicable to the real-life, co-morbid and poly-medicated patient population with osteoporosis. GPs were sceptical of guidelines and lamented the growing gap between research and clinical practice, with more frail patients ‘excluded from almost every randomised controlled trial’. They wanted ‘one message’ and guidelines to ‘reflect the reality of their practice [26, 27]’. GPs felt guidelines were too complicated, suggesting guidelines be ‘sufficiently simplistic enough’ to be useful in clinical settings [28]. Recommendations were viewed as clearer for secondary prevention and more ambiguous for primary prevention. Similar frustrations were shared by allied health and nursing with regard to fall prevention, with disparities between professions and hospital sites.

#### Sceptical of available treatments

Some HCPs doubted the efficacy of available pharmacotherapy and fall prevention programs. There was distrust of the motives of pharmaceutical companies and reported evidence from ‘sponsored’ clinical trials [25, 27]. Pharmaceutical company involvement in the provision of educational materials and supplements was also flagged by clinicians as untrustworthy. Some doctors were hesitant to prescribe a medication with silent positive health effects due to a perceived lack of tangible treatment benefit for their patients. ‘Side effects!’ were given hierarchical importance over a perceived ‘benefit that has not really been proven [20]’. Osteonecrosis of the jaw, upper gastrointestinal side effects and atypical femoral fractures were frequently cited as fearful adverse effects which outweighed potential treatment benefits. The impracticality of medication administration, particularly oral bisphosphonates and previous adverse experiences negatively shaped the decision making of GPs.

### Frustration with interdisciplinary and systemic tensions

#### Assumptions on responsibility and roles

Some hospital physicians, such as endocrinologists working in fracture liaison services (FLS), were sceptical of the ability of GPs to provide osteoporosis care due to a perceived lack of interest and knowledge [22, 28]. However, most hospital-based HCPs strongly felt ‘this has to be owned by primary care [28]’. Orthopaedic surgeons felt osteoporosis management was out of their scope of practice, stating concern that their involvement may be perceived by GPs as ‘territory infringement [29]’. On the contrary, GPs felt hospitals and specialists should take more responsibility, particularly when fractures occurred and with complex cases. To further the tension between the specialist and primary care interface, some GPs described feeling belittled in correspondence [22]. Occupational therapists felt GPs did not understand their role in osteoporotic fracture prevention [30]. Tensions existed within the hospital environment regarding fall prevention, with assumptions of the responsibility of physiotherapists by nursing and medical staff, whilst allied health voiced the importance of shared responsibility [23, 31].

#### Breakdown in communication

The inadequacy of routes of communication between different HCPs involved in osteoporosis, falls and fracture prevention was a common source of frustration hampering care. The ‘delay, it usually takes a month’, varying degrees of quality, conflicting documentation and absent communication led to a perception of fragmented care [22]. Delays in communication frequently led to osteoporosis care being overlooked. The absence of reciprocity of communication discouraged collaboration and led to a ‘very much them and us situation [28]’. HCPs were frustrated with failure in sharing of investigations and inconvenienced by the inefficiency in needing to ‘actively set out’ time to find results [29]. Whilst recommendations on osteoporosis pharmacotherapy were included in discharge summaries from hospitals, fall prevention was infrequently acknowledged in correspondence.

#### Navigating care with insufficient resources

All HCPs were dissatisfied with resource allocation at all levels of the healthcare system. The absence of adequate financial reimbursement was viewed as a disincentive to appropriate management, particularly in delivering preventative care in a system which was becoming increasingly ‘profit-focussed [22]’. GPs desired adequate reimbursement and time to ‘follow-these patients up more thoroughly [28, 32]’. When patients needed specific care, HCPs needed to negotiate management within the rules and restrictions placed by systems. This included navigating potentially prohibitive costs of investigation and treatment. HCPs also felt it was futile to address osteoporosis, falls and fracture prevention, in the absence of services for appropriate referrals, particularly allied health. Providers stressed the necessity of adequate administrative support, to promote efficiency and prevent breakdowns in communication [26, 30].

### Avoiding medical paternalism

#### Low perceived importance by patients

HCPs focussed on issues raised by patients, usually symptomatic medical problems. They perceived preventative care for asymptomatic conditions was of limited concern for patients and HCPs worried that initiating management breached a patient-centred care approach and a patient’s right to autonomy. HCPs felt the relative absence of public health campaigns for preventative care, for example compared to malignancy, contributed to patient disinterest and lack of awareness. HCPs met resistance to treatment, as ‘they (patients) can’t feel their osteoporosis’, without direct impact to their day-to-day lives [33]. Falls were considered particularly challenging as patients ‘are not going to report’ falls as they were not seen ‘as a big thing [32]’.

#### Respecting patient autonomy and dignity

HCPs thought patients viewed the prevention of falls and treatment of osteoporosis as confrontational because it signalled a degree of frailty to the patient. They identified loss of independence and fear of residential aged care placement as common concerns held by patients. Fall prevention strategies were thought to be viewed as patronising. Some GPs reflected on previous experiences, with patients ‘in denial’ or ‘dismissive [32, 34]’. Ultimately, these fears acted as a disincentive to initiating discussion in routine care. Allied health providers and nurses were hesitant to prescribe mobility aids and home modifications and seek assistance in activities of daily living due to perceived patient resistance, citing shame and denial [23, 31].

#### Minimising disease and treatment burden

HCPs, particularly GPs, were conscious of the possible negative implications of a diagnosis of osteoporosis on the psychological well-being of patients. They recognised the potential of screening to create unneeded anxiety particularly in already worried patients that would have ‘live(d) a happier life without knowing [35]’. In contrast, some GPs felt obligated to arrange screening that was not clinically indicated in ‘many healthy, very well-kept women’, who had concerns for osteoporosis [18]. Some HCPs felt defeated and forced by such obligations, deferring to patient requests for screening against their perceived clinical judgement and beyond their understanding of current recommendations.

#### Negotiating patient acceptance

HCPs recognised patients become ‘more trusting’, after demonstrating flexibility, which was necessary in building trust, confidence and rapport for longitudinal care [28]. To develop a strategy for osteoporosis, falls and fracture prevention, HCPs sometimes needed to compromise, at times accepting some suboptimal aspects of management, and adopting an ‘acceptable risk’ strategy [23]. This was particularly noted by allied health professionals when implementing fall reduction strategies, for example, with the selection of types of mobility aids or home modifications. GPs also employed shared decision making to improve treatment adherence to pharmacotherapy, including selecting options which minimised overall drug burden and discussed treatment expectations.

### Desire for improved care

#### Enhancing educational opportunities

HCPs highlighted the importance of education at all levels of training and during clinical practice. This was perceived as necessary with evolving recommendations and with increased recognition of the importance of osteoporosis, falls and fractures. Emphasis on the implications of osteoporotic fracture during formative training years was more likely to leave a lasting impression on clinicians. GPs, nurses and pharmacists ‘want to know’ and would ‘definitely go on a course’ if opportunities were available, to improve clinical confidence and ability to provide appropriate patient education [36]. Suggested educational resources included simplified checklists, coverage in medical newspapers and discussion in grand rounds, over didactic lectures. Priorities identified by clinicians included practical advice on treatment and prevention, information on locally available diagnostic services and allied health programs, which was deemed as practical, over new research.

#### Empowerment through public awareness

Increasing recognition of fracture prevention, through media exposure, was thought to be critical by HCPs to improve health-seeking behaviour in their patients. HCPs felt the general community did not recognise the link between osteoporosis, falls and fractures and its potential to reduce the quality of life. A ‘public health campaign’ could motivate individual management decisions by encouraging ‘patients (to) come in and prompt the physician and then the physician can react [37]’. HCPs felt public awareness could also empower patients to take responsibility for their bone health and improve adherence and ongoing health engagement with osteoporosis and fall prevention.

#### Accessibility through equitable healthcare

HCPs were hopeful for strategies to mitigate distance and cost barriers, particularly for those in non-metropolitan areas and the less mobile. They acknowledged the increased likelihood of these vulnerable groups ‘get(ting) lost in the system’ and suggested good public transport links and direct provision of transportation to medical appointments [28]. Due to remuneration systems in some healthcare systems, access to free GP appointments occurred at the cost of quality care. Inadvertently, patients accessing care from free ‘high-throughput, profit-focussed medical centre(s)’ were more likely to receive substandard care [22]. GPs proposed financial incentives for osteoporosis care, to mitigate inequitable quality of care.

#### Strengthening interdisciplinary collaboration

HCPs recognised a formalised team-based approach required a designated leader to coordinate care between disciplines. GPs referenced other chronic care programs, such as those existing for ‘diabetes and asthma and chronic obstructive pulmonary disease’, with specialist nurses acting as the ‘spider in the web’, and conducting the ‘groundwork [18]’. Some HCPs identified interdisciplinary hostility acting as a barrier to care and voiced the need to embrace, support and develop ‘mutual professional respect’ for the role of other HCPs in service delivery [28]. The formal multidisciplinary collaboration enabled the overcoming of these barriers, enabling clinicians to gain an appreciation for the capabilities of other professions and build positive relationships. This ultimately enabled the delivery of holistic patient-centred care and more consistent educational messaging to patients.

### Embracing the responsibility

#### Empowering patients with knowledge

Teaching patients about the consequences of osteoporotic fracture including loss of independence was perceived as a critical driver of participation in fall prevention and treatment adherence. Tailoring the approach to the individual patient, educating with the knowledge of ‘why, not just this is what’s happening’ was necessary to ensure they ‘understand what they’re trying to prevent and how it can impact on their lifestyle’ [31, 38]. Educating patients would promote health ownership and help patients to ‘remind us and even demand more from us [18]’. Clinicians recognised that patients were more receptive to education after an event, such as fall or fracture, taking the opportunity to reinforce messaging during this period. Suggestions by clinicians to supplement individualised patient education included written information, involving family and outreach sessions.

#### Overcoming misinformation

Misrepresentation of osteoporosis, particularly its pharmacotherapy, in the media led to resistance to acceptance of treatment. Debunking misconceptions and providing realistic expectations with adverse effects were accepted as part of their role as GPs. Some GPs described frustration in competing with erroneous sources of information, whilst others took pride in their clinical experience and expertise. GPs reported the need to reassure the group of patients who had excessive fear of osteoporotic fractures.

#### Providing proactive and holistic care

Most GPs took pride in their responsibility as a generalist and clinical leaders in providing continuity of care. They welcomed the opportunity to ensure their patients are in the ‘best possible situation to age well [20]’. This was perceived as necessary with the increasing burden of disease given the ageing population. They equated fracture prevention as no different to other major preventative healthcare opportunities, such as cardiovascular risk assessment, ‘I liken (10-year fracture risk assessment) to the Framingham Risk assessment [25]’. There was recognition of the detriment a fracture could have to the quality of life of their patients and that ‘osteoporosis kills more people than so many other diseases like strokes and heart attacks’ [25]. Nurses also expressed enthusiasm in sharing their fall prevention skills to patients at high risk of fracture.

## Discussion

This review found that HCPs reported facing major barriers in providing osteoporosis, falls and fracture prevention care in clinical practice, stemming from perceived patient disinterest and pre-existing beliefs on the significance of these issues within resource and time-restricted healthcare systems. There was low morale given the absence of multidisciplinary collaborative care and stunted confidence in clinical decision making given the complexities of managing the typical at-risk patient. Combined with the absence of demand from patients and concerns of treatment acceptability in the landscape of external systemic barriers, there were missed opportunities for delivering preventative care. However, many HCPs took pride in their expertise in promoting healthy ageing, longing for greater resources to support their clinical practice.

Provider perspectives of osteoporosis and related fractures varied, from a potentially catastrophic, serious disease, a simple risk factor to inconsequential and natural byproduct of ageing. GPs and surgeons, compared to specialist physicians, nurses and allied health providers, were more likely to de-prioritise osteoporotic fracture prevention compared to other co-morbidities. This is a unique perspective, which contrasts with quantitative studies, in which osteoporosis was assessed to be a preventable and important condition to primary practitioners [39]. Those who perceived osteoporotic fractures as a natural consequence of ageing were more likely to demonstrate cynicism of the utility of preventative care. This reflects deeply entrenched age discrimination in healthcare, including under-representation in medication trials, and the wider community [40]. HCPs who viewed osteoporotic fractures as a significant issue acknowledged the growing burden of disease, taking opportunities to maintain quality of life and function through the delivery of proactive care. Similarly, qualitative studies with patients have found varying perspectives on osteoporosis as a disease [41]. Similar to some HCPs in our review, some patients conceptualised fractures as traumatic, avoidable accidents from which patients eventually recover rather than potentially related to a specific diagnosis of osteoporosis [42, 43]. Public health campaigns and coverage in medical training of osteoporotic fractures as a treatable, preventable disease are required to generate recognition and interest amongst HCPs and health consumers, which in turn will promote proactive care.

Providers described hesitancy in broaching osteoporotic fracture prevention, due to the possibility of patients responding with hostility or fear. Preventive health messaging can be perceived as paternalistic and patronising, whilst provoking unwarranted anxiety in others. Contradictory to provider concerns, screening for osteoporosis has not been shown to lead to adverse emotional outcomes [44, 45]. HCPs may under-estimate patients’ perception of osteoporosis leading to reduced diagnosis and appropriate treatment for individuals with osteoporosis. Qualitative studies with patients have found that many, particularly those who have previously sustained fractures, recognised osteoporosis as a significant medical diagnosis which threatens independence and ability to work and contributes to activity avoidance [46]. Framing osteoporotic fracture prevention as a means to improve performance in activities of daily living, whilst recognising the denial and shame which may surround functional change, may increase the likelihood of recommendations being met positively by patients and enhance fracture prevention management initiation and adherence.

Limited education during training and throughout clinical practice combined with rapidly evolving guidelines contributed to low confidence in osteoporosis management, particularly amongst GPs. Although the lack of confidence surrounding pharmacotherapy was clear, there was little discussion regarding non-pharmacological aspects of osteoporotic fracture prevention, such as physical activity or nutrition in the included studies, potentially reflecting paucity of education in this area. A survey of 1153 GPs found that 88% rated teaching on osteoporosis during medical school as minimal or none [39]. Sources of misinformation, including strong media coverage on anti-resorptive adverse effects, overshadow reliable educational resources leading to scepticism and distrust of pharmacotherapy [47]. The exclusion of frailer individuals and drug company involvement in clinical trials further compounds this suspicion [48]. Despite drug-related side effects and fear of potential side effects being the most common reasons for medication discontinuation, patients describe HCPs as being frequently unable to address these information needs, directly influencing treatment adherence and their therapeutic relationship through loss of patient confidence [49, 50]. This reinforces the need for quality educational opportunities, throughout all stages of medical training and during practice, to build confidence in clinical practice, thus enabling the development of experience and the ability to provide adequate patient care and education [51].

Collaborative care for osteoporotic fracture prevention is hampered by fragmented communication and implicit hierarchy between HCPs. The negative consequences of poor communication are well-recognised in healthcare, including a breakdown of continuity of care, inconsistent treatment plans, polypharmacy and medication errors, potentially hindering patient safety [52]. Variable correspondence from hospitals post-fracture, absence of fall prevention recommendations and excess responsibility placed on patients to coordinate care contribute to delays or omission of osteoporosis fracture prevention. The inferred dominant position of doctors in the healthcare system and the hierarchy between specialists and family physicians leading to imbalanced power dynamics can curb knowledge transfer, discussions and resource allocation [53]. Patients have described frustration in navigating a system with poor interprofessional communication, recognising the tensions between primary and secondary care, which ultimately undermines the safety of patient transitions and effective care [54]. Shifting the mindset of providers away from rigid roles defined by profession, towards flexible, patient-focussed responsive care to dismantle hierarchies would promote collaboration in fracture prevention.

Despite challenges described by HCPs, many took pride in their expertise and their ability to reduce osteoporotic fracture risk. This sentiment is shared by GPs regarding all aspects of preventative care, albeit with a greater focus on the prevention of cardiovascular disease and malignancy [55, 56]. Qualitative studies have shown patients expect GPs to provide preventative healthcare, rather than focus solely on acute issues [57]. GPs however felt their enthusiasm needed to be supplemented by greater public awareness and continuing provider education. Examples of public health campaigns shown to increase public knowledge of osteoporosis include the distribution of educational materials; advertising through social media and urban platforms; and press conferences through the media [58]. Comprehensive, easily accessible and self-paced online learning resources for osteoporosis and falls, in exchange for Continuing Professional Development (CPD), may motivate clinician engagement in continuing education [59].

The themes identified in our study share similarities with other studies on the prevention of chronic disease. A qualitative study with primary care providers in managing patients with multiple chronic diseases found shared frustrations with balancing medical complexities, negotiating management within the patient’s social context and evidence-based recommendations [60]. In cardiovascular disease, patient-related barriers, including limited patient knowledge, and challenges in implementing lifestyle changes were highlighted as barriers by providers [55]. Similarities exist in providers’ scepticism and confusion with screening tools in malignancy screening [61, 62]. Unlike osteoporotic fracture prevention, there was a greater acknowledgement of mortality with cardiovascular disease and malignancy, with more GPs feeling obliged to exercise vigilance in preventative care.

This review is unique in that it covers the perspectives of various HCPs in their experience in the prevention of osteoporotic fractures, including osteoporosis as a medical diagnosis and falls as a major risk factor. The perspectives of non-medical HCPs are important given consensus recommendations of referral to allied health to reduce fall risk [63, 64]. There are limitations to our study. Only qualitative papers in the English language were included. Most studies were conducted in wealthy countries, in metropolitan settings, whereas local, population factors and cultural-specific background may influence HCPs’ perspective. The predominant patient population managed by HCPs, with educational and sociodemographic backgrounds, was not recorded in studies. Exploring perspectives of clinicians from non-English speaking backgrounds, a variety of practice settings with a range of patients from diverse backgrounds may reveal divergent perspectives. Exploration of non-English literature may have provided additional unique insights. Allied health professionals were under-represented in this review, despite being integral in falls and fracture prevention. Most medical HCPs were GPs or PCPs, with specialists, particularly those outside a FLS setting, under-represented. Most studies focussed on osteoporosis alone, when fracture prevention requires active management of major risk factors, including falls. There is the possibility of bias due to the recruitment strategy employed by several studies, which may have included HCPs with greater experience and interest in osteoporosis and falls.

Based on our review, the complex interaction between provider perspectives within the healthcare system governed by resource allocation means there will not be a ‘one size fits all’ intervention to improve care. However, a combined strategy of provider education and re-structuring of healthcare systems may facilitate improvements in osteoporotic fracture prevention. Didactic education sessions alone have not been shown to consistently improve osteoporosis management, whilst interactive educational interventions in fall prevention were associated with improved knowledge, clinical confidence and behaviour [65–67]. Re-structuring medical education should incorporate problem-based, interactive and simulation-based learning, inclusive of skill-building for interprofessional collaboration, which may provide the framework for future improvements. Educational material, succinctly outlining tailored recommendations, may further supplement formal learning sessions [68]. Structural interventions have been found to significantly improve osteoporosis management and reduce re-fracture rates [68, 69]. Multifaceted interventions, such as Fracture Liaison Services (FLS), incorporate BMD testing, education and treatment initiation. Despite robust evidence to support FLS and similar models of care, limitations exist with the breakdown in the interface with community-based primary care, accessibility for less ambulatory patients and availability in less well-resourced healthcare systems. Thus, any proposed intervention must incorporate patient demographics and local factors, including resourcing and acceptability.

## Conclusion

Our study highlights the HCP complex and varied perspectives contributing to the significant gap between the public health necessity that is osteoporotic fracture prevention. Optimal care is hampered by inadequate priority with a lack of perceived connection with illness or morbidity by HCPs, deficiencies in interprofessional collaboration and gaps in knowledge. The contextual background of low public health awareness, antagonistic views towards ageing and the ageing process and deficits in resourcing further hinder care. However, HCPs express a desire for improved care and improving expertise, which are significant for future directions. The findings support the implementation of locally relevant, multifaceted, patient-focussed, patient and provider-acceptable strategies combining education, healthcare re-structuring and shared decision making is required to bridge the osteoporosis care gap.

## Supplementary Information

Below is the link to the electronic supplementary material.

Supplementary file1 (PDF 159 KB)

Supplementary file2 (PDF 168 KB)

Supplementary file3 (PDF 151 KB)

Supplementary file4 (PDF 173 KB)

## Data Availability

Data can be made available by the researchers based on request.
