# Supplementary material for: Perspectives of healthcare providers on osteoporosis, falls and fracture risk: a systematic review and thematic synthesis of qualitative studies
Source: Arch Osteoporos. 2024 Sep 24;19(1):90. doi: 10.1007/s11657-024-01446-8 (PMC11420259; doi:10.1007/s11657-024-01446-8)
Supplement: Supplementary file 1 — Supplementary file1 (PDF 159 KB) [file 11657_2024_1446_MOESM1_ESM.pdf]

Online Resource 1: Search Strategy

A. MEDLINE (Ovid interface) (1945-present)

Search 24/05/2023

Ovid MEDLINE(R) ALL <1946 to May 24th 2023>

#	Search terms	Results
**1**	exp Osteoporosis/	62541
**2**	(osteoporo* or osteopen* or bone loss).ab,ti.	**103663**
**3**	exp Accidental Falls/	27945
**4**	(fall* or slip* or trip* or collaps*).ab,ti.	500766
**5**	exp Fractures, Bone/	207179
**6**	"fracture*".ab,ti.	252753
**7**	exp Physicians/	175559
**8**	exp General Practitioners/	10491
**9**	(doctor* or physician* or clinician* or practitioner*).ab,ti.	848513
**10**	exp Qualitative Research/	81191
**11**	exp Focus Groups/	35594
**12**	exp Interview/	29784
**13**	exp Personal Narrative/ or exp Narration/	20304
**14**	("thematic analys*" or "content analys*" or "focus group*" or ethnograph* or ethnograf* or etnograf* or "field stud*" or phenomenol* or narration* or "narrative analys*" or "narrative inquir*" or "qualitative*" or multimethodolog* or "multi-methodolog*" or "mixed method*" or "grounded theory" or "audio recording*" or "tape recording*" or audiotape* or "audio-tape*" or video* or fieldwork or "field work" or "key informant").tw.	536032
**15**	(("semi-structured" or semistructured or unstructured or informal or "in-depth" or indepth or "face-to-face" or structured or guide* or "open-ended") and (interview* or discuss* or question*)).tw.	327693
**16**	1 or 2 or 3 or 4 or 5 or 6	861449
**17**	7 or 8 or 9	923251
**18**	10 or 11 or 12 or 13 or 14 or 15	818659
**19**	16 and 17 and 18	2776
**20**	limit 19 to (english language and humans)	2525

B. EMBASE via Ovid, 1947-current

Search 240523

Embase Classic <1947 to 1973>

Embase <1974 to **May 24th 2023>**

#	Search terms	Results
**1**	exp osteoporosis/	158892
**2**	exp falling/	49267
**3**	exp fragility fracture/	23382
**4**	exp physician/	1016110
**5**	exp general practitioner/	122375
**6**	exp medical specialist/	92769
**7**	(doctor* or physician* or clinician* or practitioner*).ti.	240837
**8**	exp qualitative research/	116386
**9**	exp unstructured interview/ or exp interview/ or exp semi structured interview/	372153
**10**	exp narrative medicine/ or exp narrative/	25213
**11**	exp storytelling/	1905
**12**	exp grounded theory/	10305
**13**	exp thematic analysis/	33377
**14**	exp content analysis/	24641
**15**	exp ethnographic research/	1121
**16**	exp field study/	10001
**17**	exp phenomenology/	13671
**18**	exp qualitative analysis/	83654
**19**	(thematic analys* or content analys* or focus group* or ethnograph* or ethnograf* or etnograf* or field stud* or phenomenol* or narration* or narrative analys* or narraive inquir* or qualitative* or multimethodolog* or multi-methodology* or mixed method* or grounded theory or audio recording or tape recording or audiotap* or video*).ab,ti.	848780
**20**	(((semi-structured or semistructured or unstructured or informal or in-depth or indepth or face-to-face or structured or guide* or open-ended) and interview*) or discuss* or question* or key informant or fieldwork or field work).ab,ti.	4248682
**21**	1 or 2 or 3	214508
**22**	4 or 5 or 6 or 7	1201273
**23**	8 or 9 or 10 or 11 or 12 or 13 or 14 or 15 or 16 or 17 or 18 or 19 or 20	4976731
**24**	21 and 22 and 23	2644
**25**	limit 24 to (human and english language)	2347

C. PsychINFO via Ovid (1806-current)

APA PsycInfo Search 240523

APA PsycInfo <1806 to **May 24th 2023>**

**1**	exp Osteoporosis/	1188
**2**	(osteoporo* or osteopeni* or bone loss*).ab,ti.	2476
**3**	exp Falls/	3814
**4**	(fall* or slip* or trip* or collaps*).ab,ti.	82981
**5**	exp Grounded Theory/	4570
**6**	exp Interviews/	19305
**7**	exp Observation Methods/	6923
**8**	exp Narratives/	24956
**9**	exp Storytelling/	5928
**10**	exp Thematic Analysis/	1888
**11**	exp Content Analysis/	22033
**12**	exp Ethnography/	11011
**13**	exp Qualitative Methods/	20846
**14**	exp Participant Observation/	559
**15**	exp Phenomenology/	17922
**16**	exp Decision Making/	142991
**17**	exp Narrative Analysis/	1182
**18**	(thematic analys* or content analys* or focus group* or ethnograph* or ethnograf* or etnograf* or field stud* or phenomenolog* or narration* or narrative analys* or narrative inquir* or qualitative* or multimethodology* or multi-methodolog* or mixed method* or grounded theory or audio recording or tape recording or audiotape* or audio-tape or video* or fieldwork or field-work or key informant).tw.	449631
**19**	(((semi-structured or semistructured or unstructured or informal or in-depth or indepth or face-to-face or structured or guide* or open-ended) and interview*) or discuss* or question*).tw.	1613740
**20**	exp Physicians/ or exp Family Physicians/	48727
**21**	exp General Practitioners/	6348
**22**	(doctor* or physician* or clinician* or practitioner*).ab,ti.	298917
**23**	1 or 2 or 3 or 4	85413
**24**	20 or 21 or 22	313767
**25**	5 or 6 or 7 or 8 or 9 or 10 or 11 or 12 or 13 or 14 or 15 or 16 or 17 or 18 or 19	1958055
**26**	23 and 24 and 25	2588

**27**	limit 26 to (human and english language)	2346

D. CIHANL Complete (EBSCO interface)

#	Query	Results
**S1**	MH osteoporosis OR TI (osteoporo* OR osteopenia*) OR MH accidental falls OR MH fracture OR TI (fracture* OR broken bone*) OR osteoporotic fracture OR TI (fall* OR trip* OR slip* OR collaps*)	122551
**S2**	MH physician OR MH doctors OR MH clinician OR practitioner	123796
**S3**	MH qualitative study OR MH thematic analysis OR MH content analysis OR MH focus group OR MH ethnographic research OR MH field studies OR MH narratives+ OR MH multimethod OR MH observational method+ OR MH audiorecording OR MH semi structured interviews OR MH unstructured interview	159942
**S4**	TI ("thematic analys*" OR "content analys*" OR "focus group*" OR ethnograph* OR ethnograf* OR etnograf* OR "field stud*" OR phenomenol* OR narration* OR "narrative analys*" OR "narrative inquir*" OR qualitative* OR multimethodolog* OR "multi-methodolog*" OR "mixed method*" OR "grounded theory" OR "audio recording*" OR "tape recording*" OR audiotape* OR video* OR (("semi-structured" OR semistructured OR unstructured OR informal OR "in-depth" OR indepth OR "face-to-face" OR structured OR guide* OR "open-ended") AND (interview* OR discussion* OR questionnaire*))) OR AB ("thematic analys*" OR "content analys*" OR "focus group*" OR ethnograph* OR ethnograf* OR etnograf* OR "field stud*" OR phenomenol* OR narration* OR "narrative analys*" OR "narrative inquir*" OR qualitative* OR multimethodolog* OR "multi-methodolog*" OR "mixed method*" OR "grounded theory" OR "audio recording*" OR "tape recording*" OR audiotape* OR video* OR (("semi-structured" OR semistructured OR unstructured OR informal OR "in-depth" OR indepth OR "face-to-face" OR structured OR guide* OR "open-ended") AND (interview* OR discussion* OR question*)))	402461
**S5**	S3 OR S4	455712
**S6**	S1 AND S2 AND S5	135
