# Supplementary material for: Perspectives of healthcare providers on osteoporosis, falls and fracture risk: a systematic review and thematic synthesis of qualitative studies
Source: Arch Osteoporos. 2024 Sep 24;19(1):90. doi: 10.1007/s11657-024-01446-8 (PMC11420259; doi:10.1007/s11657-024-01446-8)
Supplement: Supplementary file 2 — Supplementary file2 (PDF 168 KB) [file 11657_2024_1446_MOESM2_ESM.pdf]

Online Resource 2: Study Characteristics

Study ID	Country	Setting	Number	Gender (M:F)	Age (years)	Clinician type	Methodological framework	Data collection	Analysis	Context/topic
Alami et al. [19]	France	Rural, semi-rural & metropolitan; public/private and mixed practices	18	8:10	Range <45->60 Median 45-60	GPs Gynaecologists Rheumatologists	Grounded theory	Semi-structured interviews and focus groups	Thematic analysis	Perspectives on osteoporosis
Allin et al. [25]	Canada	Metropolitan	22	NS	NS	GPs	Qualitative study	Semi-structured interviews	Thematic analysis	Experiences and perspectives on BMD
Bennett et al. [22]	Australia	Metropolitan	18	7:11	Mean 41 (range 28-61) (FLS clinicians) Mean 60 (range 47-72) (GPs)	Fracture Liaison Service clinicians (allied health, endocrinologists, trainees) GPs	Qualitative study	Semi-structured interviews	Inductive content analysis	Identify barriers, supports and opportunities for care following fragility fracture
Berland et al. [70]	Norway	NS	20	0:20	Range 23-56 Mean 31	Nurses	Exploratory qualitative design	Focus groups	Malterud’s modified systematic text condensation method	Experience of patient safety and falls

Chou et al. [32]	USA	NS	18	13:5	NS	GPs	Qualitative study	Semi-structured interviews	Constant comparative method of qualitative analysis of Glaser and Strauss	Perspectives and experiences on falls management
Claesson et al. [36]	Sweden	Metropolitan	13	0:13	Mean 50 Range 28-63	Nurses	Qualitative study	Focus groups	Thematic analysis	Perceptions of osteoporosis management
Drew et al. [28]	UK	Metropolitan	43	NS	NS	Nurses, orthogeriatricians, geriatricians, GPs, orthopaedic surgeons, rheumatologists	Qualitative methods	Semi-structured interviews	Thematic analysis	Perspectives on fracture prevention in hospitals
Emmett et al. [26]	UK	Metropolitan & rural	15	NS	NS	GPs	Qualitative study	Semi-structured interviews	Thematic analysis	Screening for osteoporosis
Feldstein [29]	USA	NS	57	NS	NS	GPs<br>Orthopaedic surgeons<br>Nurses<br>Pharmacists<br>Other NS	Qualitative study	Semi-structured interviews and focus groups	Thematic analysis	Perspectives and experiences of osteoporosis care
Grant et al. [21]	Australia	Metropolitan	8	4:4	NS	GPs	Qualitative study	Semi-structured interviews	Thematic analysis	Experience with falls prevention

Guzman-Clark et al. [33]	USA	Metropolitan	23	13:10	NS	Nurses, physicians	Mixed methods	Focus groups and questionnaires	NS	Management of glucocorticoid induced osteoporosis
Heng et al. [31]	Australia	Metropolitan	23	6:17	Mean 34.1	Nurses, allied health (OT, PT, allied health assistants)	Qualitative methods	Focus groups	Thematic analysis	Health professional views/perspective of delivering falls prevention programs
Iversen et al. [71]	USA	Metropolitan	12	5:7	NS	General medicine physicians, nurses	Qualitative methods	Focus groups	Thematic analysis	Perspectives of osteoporosis treatment adherence
Jaglal et al. [27]	Canada	Metropolitan	32	12:20	NS	GPs	Qualitative methods	Focus groups	Thematic analysis	Experiences, perceptions of osteoporosis
Lee et al. [23]	Australia	Metropolitan	60	15:45	GPs 51.3 Hospital doctors Acute – 27.1 Subacute – 29.5 Allied health/nursing 34.8	GPs, hospital doctors, allied health (occupational therapist, social worker, podiatrist, case managers), nurses	Qualitative methods	Focus groups & semi-structured interviews	Thematic analysis	Experiences in falls prevention

Mackenzie et al. [30]	UK	Metropolitan	11	7:4	NS	GPs, occupational therapists, physiotherapists	Qualitative methods	Semi-structured interviews	Thematic analysis	Perceptions of falls risk management
Merle et al. [20]	France	Majority metropolitan	16	11:5	Range 31-70<br>Mean 55	GPs	Qualitative study	Semi-structured interviews	Thematic analysis	Knowledge and representations of osteoporosis care and prevention
Munce et al. [73]	Canada	Metropolitan	22	12:10	NS	Family physicians	Qualitative descriptive approach as described by Sandelowski	Semi-structured interviews	Thematic analysis	BMD testing
Naik-Panvelkar et al. [38]	Australia	NS	13	8:5	NS	GPs	Qualitative study	Semi-structured interviews	Thematic analysis	Beliefs and attitudes to osteoporosis treatment
Nik et al. [37]	Malaysia	NS	19	7:12	Range 28-54	Pharmacists	Qualitative study	Focus groups & semi-structured interviews	Thematic analysis	Opinions and scopes of practice in osteoporosis
Otmar et al. [72]	Australia	Regional	16	11:5	Range 27-89	GPs, nurses	Qualitative methodology	Focus groups	Analytic or nominal comparison, derived from Mill	Beliefs and attitudes to osteoporosis and its management
Pritchard et al. [16]	Canada	NS	1054	NS	NS	Family physicians	Mixed methods	Survey (17 close ended, 2 open-ended)	Thematic analysis	Identify learning needs, barriers to implementing

										osteoporosis guidelines
Ravn Jakobsen et al. [35]	Denmark	Metropolitan	6	NS	Mean 48.7 Range 40-57 years	GPs and hospital physicians specialising in osteoporosis	Phenomenology	Semi-structured interviews	Giorgi's descriptive phenomenological method	Experience of treating asymptomatic osteoporosis
Richardson et al. [24]	UK	Metropolitan	5	NS	NS	GPs	Qualitative study	Semi-structured interviews	Thematic analysis	Beliefs and attitudes towards osteoporosis
Salminen et al. [18]	Sweden	Metropolitan	17	NS	NS	Family physicians, residents	Qualitative study	Focus groups	Thematic analysis	Views on osteoporosis management in primary healthcare
Sattar et al. [34]	Canada	Metropolitan	14	NS	NS	Oncologists	Mixed methods	Semi-structured interviews	Thematic analysis	Experience of falls
Wall et al. [17]	Canada	NS	87	NS	NS	Long term care facility physicians	NS	Survey including open ended questions	Thematic analysis	Barriers to fracture assessment and current practices to fracture risk assessment

NS: Not specified
