# Supplementary material for: Perspectives of healthcare providers on osteoporosis, falls and fracture risk: a systematic review and thematic synthesis of qualitative studies
Source: Arch Osteoporos. 2024 Sep 24;19(1):90. doi: 10.1007/s11657-024-01446-8 (PMC11420259; doi:10.1007/s11657-024-01446-8)
Supplement: Supplementary file 3 — Supplementary file3 (PDF 151 KB) [file 11657_2024_1446_MOESM3_ESM.pdf]

Online Resource 3: Consolidated Criteria for Reporting Qualitative Research (COREQ)

COREQ	Domain 1: Research team and reflexivity	Studies reporting each item	Number of studies
	Personal characteristics		
1	Interviewer/facilitator identified	[18-21, 23, 25, 27, 29-38, 70, 71, 73]	20
2	Researcher credentials	[27, 30, 32, 37, 70, 71]	6
3	Interviewer/facilitator occupation	[18, 20, 27, 33, 34, 36, 37, 70, 71]	9
5	Interviewer/facilitator research experience/training	[21, 29, 33, 36, 37, 71]	6
	Relationship with participants		
6	Relationship established prior to commencement		0
7	Participant knowledge of interviewer	[37]	1
	Domain 2: Study design		
	Theoretical framework		
9	Methodological orientation and theory	[19, 33-35]	4
	Participant selection		
10	Selection strategy	[19-28, 30-33, 35-37, 72, 73]	19
11	Method of recruitment	[18-23, 27-32, 34, 36-38, 71]	17
12	Sample size	[18-38, 70-73]	25
13	Non-participation (number/reasons)	[20, 28, 29, 32, 34, 37, 70]	7
	Setting		
14	Setting of data collection	[19, 20, 22, 23, 25, 26, 28, 32, 34, 35, 37, 38]	12
15	Presence of non-participants	[18, 36]	2
16	Sample description	[18-24, 26-31, 33, 36, 38, 70-73]	20
	Data collection		
17	Interview guide, prompts	[18-23, 25-38, 70-73]	23

18	Repeat interviews		0
19	Audio/visual recording	[18-31, 33-38, 70-73]	24
20	Field notes	[18, 20, 21, 23, 27, 30, 31, 33, 36, 37, 70, 72]	12
21	Duration	[18, 19, 21-23, 25-28, 30-38, 71-73]	21
22	Data saturation	[22, 23, 25, 27, 29, 32, 37, 71, 73]	9
23	Transcripts returned to participants	[21]	1
	Domain 3: Analysis and findings		
	Data analysis		
24	Researcher triangulation	[18-20, 22, 23, 26, 27, 30-32, 35-38, 71-73]	17
24	Number of data coders	[18-20, 22, 23, 25, 26, 28, 30-34, 36-38, 70-73]	20
25	Description of coding tree	[37]	1
27	Software	[20, 22, 23, 25, 26, 28-33, 37, 72, 73]	14
28	Participant checking	[21, 23]	2
	Reporting		
29	Participant quotations/raw data provided	[18-38, 70-73]	25
30	Data and findings consistent	[18-26, 28-38, 70-73]	25
26	Thick description (range and depth of insight into participant perspectives) provided	[18-27, 29-32, 34-38, 70-73]	23

COREQ could not be performed on two papers as a survey with open-ended questions (not focus group or interview) were the method of data collection.
