# Supplementary material for: Perspectives of healthcare providers on osteoporosis, falls and fracture risk: a systematic review and thematic synthesis of qualitative studies
Source: Arch Osteoporos. 2024 Sep 24;19(1):90. doi: 10.1007/s11657-024-01446-8 (PMC11420259; doi:10.1007/s11657-024-01446-8)
Supplement: Supplementary file 4 — Supplementary file4 (PDF 173 KB) [file 11657_2024_1446_MOESM4_ESM.pdf]

Online Resource 4: Consolidated Criteria for Reporting Qualitative Research (COREQ) criteria (by included studies)

	Personal characteristics				Relationship with participants		Theoretical framework	Participant selection				Setting			Data collection							Data analysis					Reporting			
**Study ID**	Interviewer identified	Researcher credentials	Interviewer occupation	Interviewer research experience/training	Relationship established prior to commencement	Participant knowledge of interviewer	Methodological orientation and theory	Selection strategy	Method of recruitment	Sample size	Non-participation (number/reasons)	Setting of data collection	Presence of non-participants	Sample description	Interview guide, prompts	Repeat interviews	Audio/visual recording	Field notes	Duration	Data saturation	Transcripts returned to participants	Researcher triangulation	Number of data coders	Description of coding tree	Software	Participant checking	Participant quotations/raw data provided	Data and findings consistent	Thick description (range and depth of insight into participant perspectives)	**No.* (%) COREQ criteria met**
Alami et al. [19]	•						•	•	•	•		•		•	•		•		•			•	•				•	•	•	15 (51.7)
Allin et al. [25]	•							•		•		•			•		•		•	•			•		•		•	•	•	13 (44.8)
Bennett et al. [22]								•	•	•		•		•	•		•		•	•		•	•		•		•	•	•	15 (51.7)
Berland et al. [70]	•	•	•							•	•			•	•		•	•					•				•	•	•	13 (44.8)
Chou et al. [32]	•	•						•	•	•	•	•			•				•	•		•	•		•		•	•	•	16 (55.2)
Claesson et al. [36]	•		•	•				•	•	•			•	•	•		•	•	•			•	•				•	•	•	17 (58.6)
Drew et al. [28]								•	•	•	•	•		•	•		•		•				•		•		•	•		13 (44.8)
Emmett et al. [26]								•		•		•		•	•		•		•			•	•		•		•	•	•	13 (44.8)

Feldstein et al. [29]	●			●					●	●	●			●	●		●			●					●		●	●		13 (44.8)
Grant et al. [21]	●			●				●	●	●				●	●		●	●	●		●					●	●	●	●	15 (51.7)
Guzman-Clark et al. [33]	●		●	●			●	●		●				●	●		●	●	●				●		●		●	●	●	15 (51.7)
Heng et al. [31]	●							●	●	●				●	●		●	●	●			●	●		●		●	●	●	15 (51.7)
Iversen et al. [71]	●	●	●	●					●	●				●	●		●		●	●		●	●				●	●	●	16 (55.2)
Jaglal et al. [27]	●	●	●					●	●	●				●	●		●	●	●	●		●					●		●	15 (51.7)
Lee et al. [23]	●							●	●	●		●		●	●		●	●	●	●		●	●		●	●	●	●	●	18 (62.1)
Mackenzie et al. [30]	●	●						●	●	●				●	●		●	●	●			●	●		●		●	●	●	16 (55.2)
Merle et al. [20]	●		●					●	●	●	●	●		●	●		●	●				●	●		●		●	●	●	17 (58.6)
Munce et al. [73]	●							●		●				●	●		●		●	●		●	●		●		●	●	●	14 (48.3)
Naik-Panvelkar et al. [38]	●								●	●		●		●	●		●		●			●	●				●	●	●	13 (44.8)
Nik et al. [37]	●	●	●	●		●		●	●	●	●	●			●		●	●	●	●		●	●	●	●		●	●	●	22 (75.9)
Otmar et al. [72]								●		●				●	●		●	●	●			●	●		●		●	●	●	13 (44.8)

Ravn Jakobsen et al. [35]	●						●	●		●		●			●		●		●			●					●	●	●	12 (41.4)
Richardson et al. [24]								●		●				●			●		●								●	●	●	7 (24.1)
Salminen et al. [18]	●		●						●	●			●	●	●		●	●	●			●	●				●	●	●	15 (51.7)
Sattar et al. [34]	●		●				●		●	●	●	●			●		●		●				●				●	●	●	14 (48.3)

*29 criteria assessed from the 32-point COREQ checklist
